# Viewing Strategies in Children With Visual Impairment and Children With Normal Vision: A Systematic Scoping Review

**DOI:** 10.3389/fpsyg.2022.898719

**Published:** 2022-06-17

**Authors:** Anke Fonteyn-Vinke, Bianca Huurneman, Frouke N. Boonstra

**Affiliations:** ^1^Royal Dutch Visio, Nijmegen, Netherlands; ^2^Behavioral Science Institute, Radboud University, Nijmegen, Netherlands; ^3^Department of Cognitive Neuroscience, Donders Institute for Brain, Cognition and Behaviour, Radboud University Medical Centre, Nijmegen, Netherlands

**Keywords:** viewing strategies, (cerebral) visual impairment, visual rehabilitation, reading strategies, gaze strategies, search strategies

## Abstract

Viewing strategies are strategies used to support visual information processing. These strategies may differ between children with cerebral visual impairment (CVI), children with ocular visual impairment, and children with normal vision since visual impairment might have an impact on viewing behavior. In current visual rehabilitation practice a variety of strategies is used without consideration of the differences in etiology of the visual impairment or in the spontaneous viewing strategies used. This systematic scoping review focuses on viewing strategies used during near school-based tasks like reading and on possible interventions aimed at viewing strategies. The goal is threefold: (1) creating a clear concept of viewing strategies, (2) mapping differences in viewing strategies between children with ocular visual impairment, children with CVI and children with normal vision, and (3) identifying interventions that can improve visual processing by targeting viewing strategies. Four databases were used to conduct the literature search: PubMed, Embase, PsycINFO and Cochrane. Seven hundred and ninety-nine articles were screened by two independent reviewers using PRISMA reporting guidelines of which 30 were included for qualitative analysis. Only five studies explicitly mentioned strategies used during visual processing, namely gaze strategies, reading strategies and search strategies. We define a viewing strategy as a conscious and systematic way of viewing during task performance. The results of this review are integrated with different attention network systems, which provide direction on how to design future interventions targeting the use of viewing strategies to improve different aspects of visual processing.

## Introduction

The etiology of visual impairment in childhood is very heterogeneous. Visual impairment in children is caused by ocular diseases and/or genetic factors affecting the eye or by cerebral visual impairment (CVI). CVI comprises visual malfunction due to retro-chiasmal and visual association pathway pathology (Philip and Dutton, 2014). In Western countries CVI is the most prevalent cause of visual impairment (Fazzi et al., 2007; Kran et al., 2019). Since the etiology of visual impairment in children is diverse, a variety of visual problems can occur and visual rehabilitation can focus on different stages of visual processing. For example, deficits in lower visual functions, like visual acuity, visual field or contrast sensitivity, are often compensated by the use of aids or adjustments to the environment. On the other hand, deficits in higher order visual functions can be trained (e.g., strategic eye movement training). What remains unclear in the field is how children implement viewing strategies. The aim of this review is to provide an overview of the viewing strategies used during near school-based tasks, such as reading, in children with and without (C)VI and available interventions.

Below, four antecedents leading to this review are elaborated: (1) the benefits of compensatory strategies in subjects with brain damage, (2) in current visual rehabilitation a variety of strategies is used without consideration of the different etiologies of visual impairment, (3) viewing strategies hold potential benefits for visual attention, and (4) oculomotor behavior provides insights into viewing strategies.

The diversity of visual deficits in CVI is considerable, and the impact of CVI on education and rehabilitation strategies is less well-understood than for ocular visual impairments. A recent review states that educational strategies employed for children with ocular impairments are largely ineffective for children with CVI (Martin et al., 2016). There is evidence for cross modal brain reorganization in blind individuals as demonstrated by robust activation in the occipital cortex while performing non-visual tasks (Kujala et al., 2000; Fine and Park, 2018). Still, an absence of vision during the critical period seems to weaken auditory and tactile spatial representations (Pasqualotto and Newell, 2007; Finocchietti et al., 2015; Cappagli et al., 2017), while temporal tactile judgements can be superior in congenitally blind subjects (Roder et al., 2004). There is little research on compensatory mechanisms in CVI. Bryck and Fisher (2012) mention that strengthening compensatory or strategic processes can support information processing in children with acquired cerebral damage. An example of such a strategy is teaching self-verbalization to help guide action. Another example of strategic processes in rehabilitation is the use of eye movement training in adults after stroke (resulting in visual field deficits or neglect). Compensatory strategies can involve visual imagery, visual search strategies and compensating eye movements to improve visual search time and accuracy as well as to improve attention and daily living activities. These compensatory processes increase brain activation reflecting neural processes supporting task performance (Bryck and Fisher, 2012).

Since the etiology of CVI is located in the brain, visual training might target the use of viewing strategies to guide visual behavior. In current clinical rehabilitation practice, viewing strategy training is commonly employed. A viewing strategy can be defined as the systematic way in which a visual task is consciously approached. Optimally, the chosen strategy fits the task at hand and contributes to accurate and fast visual information processing. Yet, little is known about viewing strategies used by children with (cerebral) visual impairment. Based on the variety of causes of (cerebral) visual impairment and clinical practice it would be conceivable that children with CVI use different viewing strategies compared to children with ocular visual impairment and children with normal vision. Knowledge about these strategies is needed to adapt rehabilitation techniques and training to the different problems in visual functioning.

CVI is most often accompanied by impairments in higher order visuospatial processing, visual selective attention, and object recognition (Bennett et al., 2020; Zuidhoek, 2020). Targeting visual attention is likely to have a widespread beneficial impact on multiple aspects of higher and lower level stages of visual processing. A commonly accepted framework of attention networks has been presented by Posner (Petersen and Posner, 2012). This framework suggests that there are three attentional systems in the human brain: an alerting network (which focuses on brain stem arousal systems along with right hemisphere systems related to sustained vigilance), an orienting network (involving among other regions, the parietal cortex and frontal eye fields, related to the ability to direct visual attention and prioritize sensory input by selecting a modality or location) and an executive network (including the midline frontal/anterior cingulate cortex related to the ability to direct focal attention and cope with interference). Viewing strategies are plans that are consciously addressed before starting a visual task to facilitate achieving a desired goal (e.g., accuracy or speed up performance). These strategies can put a demand on different aspects of the attention networks. For example, a viewing strategy can involve instructing children to look in a structured way (i.e., left to right during reading) while keeping their attention directed at the task at hand (e.g., by using a warning signal or check points). This strategy is directed at the alerting system. An example of a viewing strategy directed at the orienting system is a strategy in which a child selects a visual array fit to the task at hand: i.e., a larger part of the picture for global processing in scene interpretation or zooming in on details (local processing). Finally, an example of a viewing strategy which puts a large demand on the executive network is a strategy in which the child focusses on a specific target which restricts the perceptual awareness of surrounding targets.

Williams et al. (2011) showed that problems in higher level visual functions, especially difficulties with interpreting cluttered scenes (visual selective attention) and visuomotor functions, are associated with underachievement in reading and in mathematics. Children with CVI demonstrate more random visual search patterns (Kooiker et al., 2016), which may be associated with less strategic visual processing. Results from a review in dyslexic and normal readers (Kulp and Schmidt, 1996) indicate that during primary school years oculomotor skills improve and are associated with increases in reading rate and ability. For example, fixation duration decreases, saccade length increases, the number of regressions (backward saccades) decreases and the perceptual span decreases during the primary school years. The authors concluded that accurate eye movements are integral to achieving reading proficiency. Some visual impairments are accompanied by oculomotor abnormalities, such as nystagmus (i.e., involuntary bilateral oscillating eye movements). Thomas et al. (2011) demonstrated that individuals with infantile nystagmus (IN) on average show slightly lower reading speeds than controls, but that there are also individuals with IN showing higher reading speeds than normal controls despite their foveal and ocular deficits. Adults with IN use several strategies to manipulate their nystagmus during reading, such as: (1) suppression of corrective quick phases allowing involuntary slow phases to achieve the desired goal, (2) voluntarily changing the character of the involuntary slow phases using quick phases, and (3) correction of involuntary slow phases using quick phases. This research provided evidence that accurate eye movements might not be as integral as once thought to reach good reading speeds and demonstrated that individuals with oculomotor deficits can adapt to and compensate for their condition by using strategies with which they can reach (near) normal reading speeds.

The threefold goal of this scoping review is: (1) to provide a clear concept of viewing strategies, (2) to map differences in viewing strategies between children with ocular visual impairment, children with CVI and children with normal vision and (3) to identify interventions that can improve visual processing by targeting viewing strategies.

## Methods

### Search Strategy

Studies were identified through electronic databases searching in PubMed, EMBASE, PsycINFO and Cochrane Library. The final search was run on 20 December 2020. In addition to the articles found with our search query, reference lists of included articles were scanned and experts were consulted. No limitation regarding year of publication was applied. The search was developed by an experienced clinical librarian. The selection was made by the first two authors of the article. The following search strategy was used to search for all databases using MeSH terms and keywords: visual perception (MeSH), viewing strategy or viewing skills, reading strategy or reading skills, visual processing or perceptual exploration or perceptual processing or fixation strategy or perceptual learning or visual development. An example of the search strategy used in PubMed is shown in Table 1.

**Table 1:** Search history in PubMed.

**Search**	**Most recent queries**
#6	#4 AND #5
#5	“Video Games” [MeSH] OR “Education”[MeSH] OR Training OR Game* OR Intervention* OR rehabilitation
#4	#1 AND #2 AND #3
#3	“Visually Impaired Persons” [MeSH] OR “Vision, Low” [MeSH] OR “Vision Disorders” [MeSH] OR Visually impaired OR visual impairment OR CVI OR low vision.
#2	Child [MeSH] OR child* OR schoolchild* OR pediatri* OR paediatr* OR boy OR boys OR boyhood OR girl OR girls OR girlhood OR youth OR youths
#1	Visual Perception [MeSH] AND (viewing strategy OR viewing skill* OR viewing difficult* OR reading strateg* OR reading skill* OR reading difficult* OR visual processing OR perceptual exploration* OR perceptual processing OR fixation strateg* OR perceptual learning OR visual development)

### Study Selection

Two authors (AF and BH) independently reviewed the list of results and identified relevant articles, based on predefined in- and exclusion criteria regarding viewing strategies of school-aged children. The inclusion criteria are presented in Table 2. All studies concerning viewing strategies, viewing skills, reading strategies and efficiency, accuracy or speed of visual processing or ways to train viewing behavior (strategy, interventions, visual training using strategies) were included. Articles involving non-school aged children were excluded (subject <4 or >12 years), because the focus in this review is on children and developmental stage affects viewing behavior. Also, studies about children with intellectual disabilities, children with psychiatric disorders (DSM related diagnosis like ADHD and ASS), and children with amblyopia were excluded, to create a clear picture of viewing strategies in typically developing children vs. children with (cerebral) visual impairment. An exception was made for studies in the above mentioned groups if the study contained information about visual viewing strategies used by a typically developing control group, in this case studies were included. Animal or machine studies, studies concerning medical diagnosis (for example describing imaging techniques like PET/MEG/MRI/CT) and studies aimed at viewing behavior regarding moving stimuli were excluded. We decided to exclude studies with moving stimuli, because we expect that different viewing strategies are used during tasks with moving stimuli compared to school-based tasks. Disagreements during selection were resolved by application of criteria, discussion and consensus.

**Table 2:** Inclusion criteria.

**Inclusion criteria**	
Population	• Children with ocular visual impairment 4–12 years • Children with cerebral visual impairment 4–12 years • Children with normal vision 4–12 years
Intervention	• (Longitudinal) Cohort • Cross sectional • Randomized controlled trials • Non-randomized controlled trials
Comparison	• Differences in viewing strategies between (age) groups • Training of viewing strategies
Outcome measurements	• Description of viewing strategy • Temporal or spatial aspects of visual processing (e.g., visual acuity, speed) • Reading strategies • Viewing strategy interventions

Included articles focused on the concept of viewing strategies or viewing behavior during near school-based tasks in a variety of children. Since limited research about children with (cerebral) visual impairment is available, we choose to include articles describing viewing behavior of children with normal vision (i.e., studies describing control group data). For example we included articles concerning reading strategies by disabled or dyslexic children and controls. Control data provide information about viewing strategies used in children with normal vision which makes their inclusion relevant for this scoping review. On consensus five articles regarding the use of Irlen filters were included, since the use of aids may count as a strategy used by the visually impaired child. Since visual training programs commonly are assigned to children in primary school, the age was limited to 4–12 years old.

### Quality Assessment

The included studies appeared very heterogeneous in subjects, paradigm and outcome measures. There was too little information provided on quantitative outcome measures regarding viewings strategies to conduct a meta-analysis. Therefore, the results of the studies will be described in a narrative manner. To assess the quality of the articles included in this scoping review, we modified the QUADAS tool (Whiting et al., 2011) for the non-intervention studies to fit it to our review purposes (Table 3). For the intervention studies we used the Cochrane Collaboration's tool for assessing risk of bias (see Supplementary Tables 4a,b for the results of the quality assessment).

**Table 3:** QUADAS criteria and modifications used in the current study.

**Original QUADAS Whiting et al. (2011)**	**Modified QUADAS**
1. Was the spectrum of patients representative of the patients who will receive the test in practice?	This question was not applicable. The scoping review focused on three target groups. The studies were only included if fitted to the target groups.
2. Were selection criteria clearly described?	This question was unmodified.
3. Is the reference standard likely to correctly classify the target condition?	This question was not applicable.
4. Is the time period between reference standard and index test short enough to be reasonably sure that the target condition did not change between the two tests?	This question was not applicable. We did not compare results obtained with a reference and index test.
5. Did the sample or a random selection of the sample receive verification using a reference standard of diagnosis?	This question was not applicable.
6. Did patients receive the same reference standard regardless of the index test result?	This question was not applicable.
7. Was the reference standard independent of the index test (i.e., the index test did not form part of the reference standard?)	This question was not applicable.
8. Was the execution of the index test described in sufficient detail to permit replication of the test?	We modified the questions 8 and 9 to a more general question regarding test procedure. Was the execution of the test procedure described in sufficient detail to permit replication of the test?
9. Was the execution of the reference standard described in sufficient detail to permit its replication?	See 8.
10. Were the index test results interpreted without knowledge of the results of the reference standard?	This question was not applicable.
11. Were the reference standard results interpreted without knowledge of the results of the index test?	This question was not applicable.
12. Were the same clinical data available when test results were interpreted as would be available when the test is used in practice?	This question was not applicable. We did not aim at one particular reference test.
13. Were uninterpretable/intermediate test results reported?	This question was unmodified.
14. Were withdrawals from the study explained?	This question was unmodified.

## Results

### Results of Search and Selection Process

**Figure 1:**
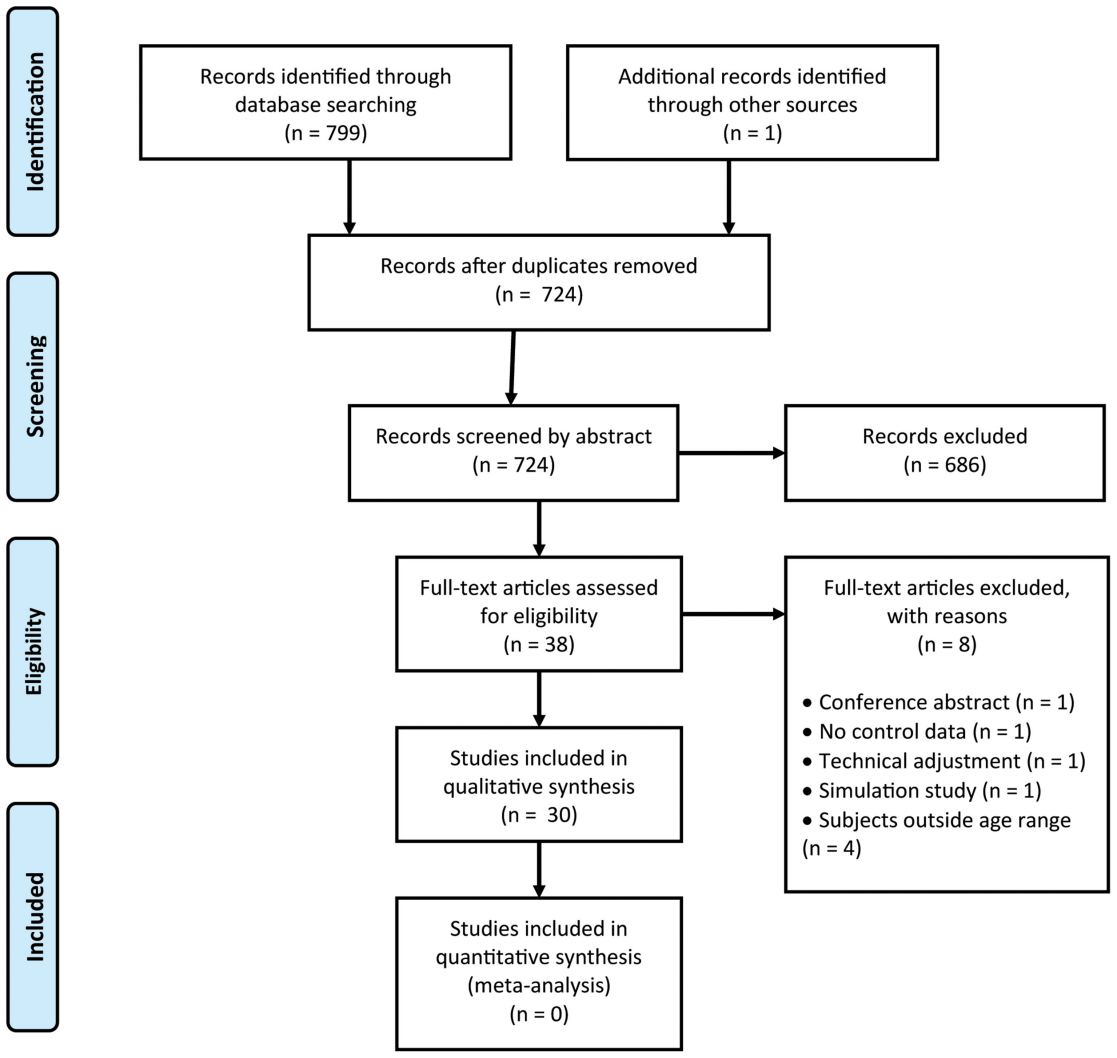
PRISMA 2009 Flow Chart.

The conducted search of PubMed, EMBASE, PsycINFO and Cochrane Library provided a total number of 799 articles. Duplicates were removed (*n* = 75) and 724 articles were screened by abstract. In addition one article was identified by an expert (Barsingerhorn et al., 2018). A total of 686 articles were discarded for not meeting inclusion criteria. Of these articles 400 articles did not contain our primary outcome measures, 123 articles included children with additional impairments (intellectual disabilities, amblyopia, psychiatric disorders) and no control data and 84 articles only concerned medical diagnosis. Another 41 articles were excluded because the age of the subjects fell outside the primary school years (<4 or > 12 years). After full inspection eight articles were discarded (see PRISMA Flow Chart Figure 1). The remaining 30 quantitative studies consisted of 12 non randomized controlled trials (Non-RCT), 10 cross sectional studies, two cohort studies, and six case control studies.

### Description of the Included Studies

Thirty-three studies were included. None of the included articles described viewing strategies as an outcome measure. In order to facilitate comprehensibility we categorized the included studies in the following manner: (1) studies regarding viewing strategies, (2) studies describing viewing behavior in children with normal vision, (3) studies describing viewing behavior in children with (cerebral) visual impairment, and (4) intervention studies focused at improving viewing behavior. We found five studies regarding viewing strategies (Robinson and Conway, 1994; Robinson and Foreman, 1999; Wilkinson et al., 2008; Pollux et al., 2014; Vinuela-Navarro et al., 2017). None of these five studies involved children with (C)VI.

Fourteen of the included studies were focused on viewing behavior in children with normal vision. A large part of these articles described differences between good readers and children with reading difficulties. Three studies were found which evaluated viewing behavior in children with (C)VI. Finally, thirteen studies were found which evaluated the effectiveness of behavioral treatment on visual processing proficiency in children with normal vision and/or children with (C)VI. The characteristics and outcomes of these studies are presented in Supplementary Tables 1–3. The quality assessment is presented in Supplementary Table 4a (non-intervention studies) and Supplementary Table 4b (intervention studies).

#### Viewing Strategies

A variety of viewing strategies were mentioned in five studies. None of these five studies involved children with (cerebral) visual impairment. The tasks for which viewing strategies were described varied across these studies: three studies mentioned strategies during reading (Robinson and Conway, 1994; Robinson and Foreman, 1999; Vinuela-Navarro et al., 2017), one study described gaze strategies during categorization of subtle facial expressions (Pollux et al., 2014) and one study described search strategies during symbol discrimination (Wilkinson et al., 2008). Study characteristics and descriptions of viewing strategies mentioned are presented in Table 4.

**Table 4:** Study characteristics and descriptions of viewing strategies.

**Reference**	**Type of study**	**Number of participants, group**	**Age**	**Method**	**Category**	**Description of viewing strategy**
Pollux et al. (2014)	Cohort Study	• Children with normal vision (*N* = 16) • Adults with normal vision (N = 16, not further regarded)	8;2–9;3 years	*Intervention:* Four training sessions on four consecutive days with a self-paced, free-viewing facial expression categorization task using emotional faces with varying intensity levels *Outcome measures:* • Behavioral measures (accuracy, response times, incorrect response) • Eye movement measures (number of fixations, proportion of fixations and viewing times on different facial features, proportion of fixations and viewing times of different facial features during second fixation)	Gaze strategy	To categorize subtle facial expressions, a holistic gaze strategy is necessary to extract relevant facial cues from all internal features.
Robinson and Conway (1994)	Non-RCT	• Experimental group • *N* = 29 children with reading or study problems • Control group • *N* = 31 children with similar reading and learning problems as the experimental group (age matched)	9–14 years	*Intervention:* Four months of Irlen filter (color overlays) use *Outcome measures:* - Questionnaire relating to reading and writing performance - series of visual tasks	Reading strategy	Reading strategies in poor readers (based on analyses of reading errors) involved: 1. guessing words from single-letter cues 2. rereading of lines 3. skipping words or lines
Robinson and Foreman (1999)	Non-RCT	• Experimental group • *N* = 113 children with reading difficulties • Control group • *N* = 35 children with children with reading difficulties (age matched)	9–13 years	*Intervention:* initial test, placebo tint (4 months), diagnosed tint (8 months) and diagnosed tint (20 months after baseline). *Outcome measures:* - Questionnaire relating to reading and writing performance - series of visual tasks and assessment	Reading strategy	See above.
Vinuela-Navarro et al. (2017)	Case control	• *N* = 120 children without delayed reading skills • *N* = 43 children with delayed reading skills	4–11 years	*Outcome measures:* - main sequence (collected with the Tobii TX300 eye tracker by showing children cartoon characters horizontally from-20° to +20° in steps of 5°). - Fixation stability (by showing children an animated stimulus in the center of the screen for 8 s). - Saccade number - Saccade amplitude	Reading strategy	Good readers showed a similar eye movement strategy for each line of text during reading (number of saccades, fixations and duration of fixations were comparable) Poor readers performed very differently in each line (unstructured and disorganized eye movement strategy).
Wilkinson et al. (2008)	Cross sectional	• *N* = 10 children with Down Syndrome (DS) • *N* = 8 typically developing children > 4 years (TDO) • *N* = 8 typically developing children <4 years (TDY)	DS: 106–201 months; TDO: 48–57 months; TDY: 40–46 months;	Line drawings of symbols in two color conditions (clustered and distributed arrays). *Three tasks:* 1. auditory—visual matching of food stimuli 2. visual—visual matching of clothing stimuli 3. visual—visual matching of activity stimuli *Outcome measures:* - Accuracy (percentage correct) - speed (RT)	Search strategy	Color cueing facilitates visual search for symbols.

Vinuela-Navarro et al. (2017) explicitly addressed oculomotor strategies used by children with and without reading problems. Children without reading problems showed similar eye movement strategies for each line of text (similar number of saccades and fixation durations) while children with reading problems showed a disorganized oculomotor strategy which differed per line [more fixations, extended fixation duration (progressive and regressive)].

In the two studies by Robinson and Conway (1994); Robinson and Foreman (1999) reading strategies are deduced by type of reading errors made by children with reading problems, namely pauses and refusals (grapheme substitutions or semantic substitutions). Based on an oral reading error analysis, the findings indicate that the use of an Irlen lens decreases the number of pauses and increases reading rate. The authors conclude that enhanced print clarity may facilitate the use of rereading as a strategy for monitoring word recognition thereby enhancing reading comprehension.

Pollux et al. (2014) investigated gaze distributions in facial expression categorization. In this study participants (adults and 9-year-olds) received feedback directly after the categorization of a facial expression with a variety of meanings (happy—sad—fearful). Eye movements were measured during the first and fourth training session. This study showed a holistic gaze strategy is used to extract relevant facial cues from all internal features when categorizing subtle expressions. More holistic processing is needed to categorize subtle expressions more accurately.

Wilkinson et al. (2008) addressed search strategies in a study regarding the use of a visual communication array for children with Down Syndrome. The reaction times to visual symbols in an array were measured for typically developing children and children with Down Syndrome. Both groups showed shorter reaction times on a verbal or visual cue to find the target symbol in an array when symbols were clustered by color, indicating that color cueing facilitates visual search and can be used as a visual strategy to enhance reaction times.

#### Viewing Behavior: Normal Vision

Fourteen studies regarding viewing behavior in school-based tasks—mainly reading—in children with normal vision were included (see Supplementary Table 1). One study evaluated the relationship between optometric parameters, e.g., refractive error and binocular vision measures, and reading skills (Vinuela-Navarro et al., 2017). The authors found no relationship between optometric measures (visual acuity, refractive error, ocular alignment, convergence, stereopsis and accommodation accuracy) and reading skills. Riddell et al. (1990) compared children with good and poor vergence control in spatial localization skills. Children with unstable vergence had trouble localizing small objects in visual space, which the authors contributed to problems maintaining an accurate visuospatial map. For reading, the ability to localize letters within a word might be impaired in children with poor vergence control.

Oculomotor measure comparisons between average and poor readers were collected in three studies (Medland et al., 2010; Nilsson Benfatto et al., 2016; Vinuela-Navarro et al., 2017). These studies demonstrated that: (1) average readers required fewer fixations while reading a line of text than children with reading difficulties, while fixation stability was found to be comparable (Vinuela-Navarro et al., 2017), (2) in average readers, fixation durations (progressive and regressive) were shorter compared to children with reading problems (Nilsson Benfatto et al., 2016; Vinuela-Navarro et al., 2017), (3) saccade amplitudes were greater for children without than with reading problems (Nilsson Benfatto et al., 2016), despite normal saccadic main sequences in children with reading problems (Vinuela-Navarro et al., 2017), and (4) children without reading problems showed faster reading times for the habitual reading direction (left to right in English readers vs. right to left for Arabic readers) (Medland et al., 2010). The studies by Vinuela-Navarro et al. (2017) and Medland et al. (2010) demonstrated that oculomotor functions are integral to reading processes, but do not give insights into causality (i.e., whether reading problems result in abnormal eye movements or whether abnormal eye movements result in reading problems).

Visual attention was measured in two studies comparing normal and dyslexic readers (Solan et al., 2007; Franceschini et al., 2017). Franceschini et al. (2017) provided evidence for differences in visual attentional processes between children with dyslexia and normal controls. In the study by Franceschini et al. (2017), five behavioral experiments provided evidence for global perception as predictive of reading skills. Solan et al. (2007) evaluated the relation between reading comprehension, visual attention and magnocellular processing (as measured with a coherent motion task) in Grade 7 students (19 good readers and 23 poor readers). Group differences were found on all measures. The authors concluded that diagnostic batteries for students who have been identified as reading disabled should include magnocellular and visual attention tests.

The relation between visual temporal processing and reading performance was evaluated using spatial visual stimuli (dot counting) in the study by Eden et al. (1995). Three groups of 5^th^ graders were compared with regards to performance on the temporal and spatial dot counting task: children with normal vision (*n* = 39), reading disabled children (*n* = 26), and backward reading children (*n* = 12). The control group performed better on the temporal dot counting task than reading disabled children, while there were no group differences on the spatial dot counting task. This study emphasizes the relationship between visual temporal processing skills and reading performance. Spatial abilities might however, play a role in predicting reading ability in young children. Fisher et al. (1985) found evidence for a relation between visuospatial abilities (lef-right coding) and (pre)reading skills in 4–7 year old children (Fisher et al., 1985).

The relation between visual learning and reading skills for children with and without reading problems was evaluated by Tong et al. (2019) and Garcia et al. (2019). Both studies showed that children without reading problems are better in learning of structures in visual elements (visual statistical learning, Tong et al., 2019) and fixed combinations (fixed bindings in four element non-sense-words and -shapes, Garcia et al., 2019) compared to children with reading problems. Visual statistical learning—the ability to unconsciously extract and integrate the structure of various (visual or auditory) elements to produce a unitary structure for further learning—appeared to be a significant predictor of Chinese word reading (Tong et al., 2019).

Lutzer (1986) measured color discrimination in children with varying intellectual capacities. This study showed no difference in the ability to match-to-sample colors after training in ranking colors vs. match-to-sample training. Children with lower cognitive abilities made more errors in color discrimination compared to average and gifted children.

In sum, oculomotor studies demonstrate that: (1) average readers use less fixations and regressions during reading, and (2) a shorter fixation duration found in average readers is linked with greater amplitudes of saccades during reading. Eye movements take a developmental leap when children learn to read more fluently, but are not essential to learn to read. Visual attentional processes seem to influence reading performance as well: average readers more often used global before local processing and performed better on visual temporal processing tasks (like dot counting and coherent motion) compared to children with reading problems.

#### Viewing Behavior: (Cerebral) Visual Impairment

Three studies were included with viewing behavior as an outcome measure in children with (C)VI (see Supplementary Table 2). These studies evaluated viewing behavior in children with (C)VI and children with normal vision (Kooiker et al., 2014, 2015; Barsingerhorn et al., 2018). Barsingerhorn et al. (2018) used a speed acuity test in which children with (C)VI were asked to indicate the orientation of a Landolt C symbol for a range of acuities surrounding their threshold acuity. Both children with ocular VI as well as children with CVI showed longer reaction times to the visual symbols compared to controls. Children with ocular pathology were 170 ± 28 (SD) ms slower than children with normal vision; children with CVI were 232 ± 36 (SD) ms slower. In addition, reaction times for children with ocular VI and children with CVI were also longer for simple visual and auditory detection tasks. This might refer to a more general underlying problem in sensorimotor functioning. Kooiker et al. (2014, 2015) measured reaction to fixation times while presenting a cartoon image on the screen. Quantitative measurement of orienting responses showed longer reaction times for children at risk for CVI and with the clinical diagnosis of CVI compared to typically developing children. Overall, the reaction times on cartoon stimuli were the shortest followed by contrast and form. Kooiker et al. showed an increased fixation area on cartoons in children with low visual acuity and children with nystagmus compared to normal controls.

Overall, children with (C)VI showed longer reaction times to visual stimuli than controls. In addition, children with low visual acuity and children with nystagmus showed increased gaze fixation areas on cartoons.

#### Interventions

Twelve non-randomized controlled trials (non-RCT) were included in this review, and one cohort study (see Supplementary Table 3). Seven of the non-R-CT studies involved children with (C)VI (Obrzut et al., 1982; Huurneman et al., 2013, 2016a,b,c, 2020; Yu et al., 2020). Five intervention studies were focused on children with reading problems versus controls (Bieger, 1974; Robinson and Conway, 1994; Robinson and Foreman, 1999; Hall et al., 2013; Zhao et al., 2019).

One training program was focused on improving visual processing skills in children with poor visual processing (Obrzut et al., 1982). The training program, “Learning to Look and Listen,” consists of three sections aiming at viewing strategies: (1) hierarchical analysis (part vs. whole), (2) systematic scanning and (3) dimensional differences. After visual information processing training, performance on visual perceptual tasks as the Bender Gestalt Closure task was improved for the training group vs. a contrast and a control group The training group maintained their progress 5–6 weeks after the training. However, academic performance did not change after training.

Three studies used visual training programs to improve reading skills (Bieger, 1974; Huurneman et al., 2016b; Zhao et al., 2019). One of these studies was conducted in children with visual impairment (i.e., infantile nystagmus) (Huurneman et al., 2016a,b,c). Huurneman et al. (2016b) compared reading performance in children with infantile nystagmus (IN) and controls. Maximum reading speed and acuity reserve did not differ between both groups. However, reading acuity and critical print size were larger for children with IN than for normal controls. After ten computerized crowding training sessions, reading acuity improved as well as critical print size. The results of the reading study indicate that not only visual acuity, but crowding is also related to reading performance in children with IN. Also, training on a computerized crowded letter discrimination task can contribute to improvements in reading performance. The study by Bieger (1974) showed no improvement on the ability to discriminate single words after visual training (i.e., Frostig program plus visual components). Although non-readers with perceptual problems improved on perceptual skills after visual training, reading skills did not improve. Zhao et al. (2019) created different research groups based on visual attentional span (VAS) intact or dysfunctional, and reading performance (dyslexic vs. normal readers). For children with dyslexia with VAS dysfunction visual training not only improved VAS function, but reading performance as well. Children with dyslexia with intact VAS did not improve on reading performance after VAS based training. VAS based training included bottom-up attention (length estimation task), top-down attentional modulation (visual search and digit canceling) and eye movement control (visual tracking).

Three studies evaluated the effect of the use of colored overlays. These studies showed inconsistent evidence that colored overlays improve reading performance (Robinson and Conway, 1994; Robinson and Foreman, 1999; Hall et al., 2013).

Huurneman et al. conducted a variety of studies on perceptual learning in children with visual impairment (Huurneman et al., 2013, 2016a,b,c, 2020). Perceptual learning is shown to improve visual functions like near visual acuity, stereopsis and crowding for children with visual impairment and these improvements are retained over time (Huurneman et al., 2020). In their first study comparing different types of pen-and-paper perceptual learning paradigms (i.e. a magnifier group, uncrowded letter training group and crowded letter training group, Huurneman et al., 2013), task-specific improvements were shown in all groups. Only the crowded perceptual learning group showed transfer to crowded near visual acuity (Huurneman et al., 2013). The improvements in near visual acuity after a pen-and-paper visual training did not transfer to distance visual acuity or fine motor skills (Huurneman et al., 2020) after six weeks. Improvements in distance visual acuity and reading, indicating broad learning transfer, were shown after a computerized crowded letter discrimination training (Huurneman et al., 2016a,b,c). A study by Yu et al. (2020) indicates that perceptual learning can be improved by the use of electronic visual devices (5–10× magnification) for children with moderate to severe visual impairment.

In sum, although different visual processing skills improved after training (i.e., gestalt closure, reading acuity), effects on academic outcome measures are often not reported or absent (Obrzut et al., 1982). The extent of learning transfer seems to depend on the training paradigm that is used. The use of colored overlays to improve detailed word analysis showed varying results. Computerized crowded letter discrimination training and a VAS based training showed potential positive effects on reading performance. Perceptual learning was shown to improve visual functions like near visual acuity and crowding for children with visual impairment and improvements seem to be retained over time. For moderate to severe visual impairment additional use of electronic aids might boost the effect of perceptual learning paradigms.

## Discussion

In this scoping review we focused on viewing strategies used during near school-based tasks and on possible interventions targeting viewing strategies for children with (cerebral) visual impairment. The main goals of this scoping review were to define a concept of viewing strategies and to compare viewing strategies between children with normal vision and children with (cerebral) visual impairment. In this scoping review we found no published research regarding viewing strategies for (cerebral) visually impaired children which makes a comparison between groups impossible. The lack of published research illustrates a giant gap between daily child vision rehabilitation practice, in which viewing strategies are a key component, and scientific evidence. Even for school-aged children with normal vision, literature about viewing strategies is scarce.

To create a concept of viewing strategies, information could be extracted from a total number of five studies in which the use of viewing strategies is mentioned (Robinson and Conway, 1994; Robinson and Foreman, 1999; Wilkinson et al., 2008; Pollux et al., 2014; Vinuela-Navarro et al., 2017). In the introduction of this paper, viewing strategy was described as a conscious and systematic way of approaching a task. Since the use of the term viewing strategies was sparse, all descriptions of more implicit or unconscious strategies were included in this review. Viewing strategies appear to be task-dependent. Within the broader concept of viewing strategies a differentiation seems necessary dependent on the visual task at hand: gaze strategies, reading strategies and search strategies.

**Figure 2:**
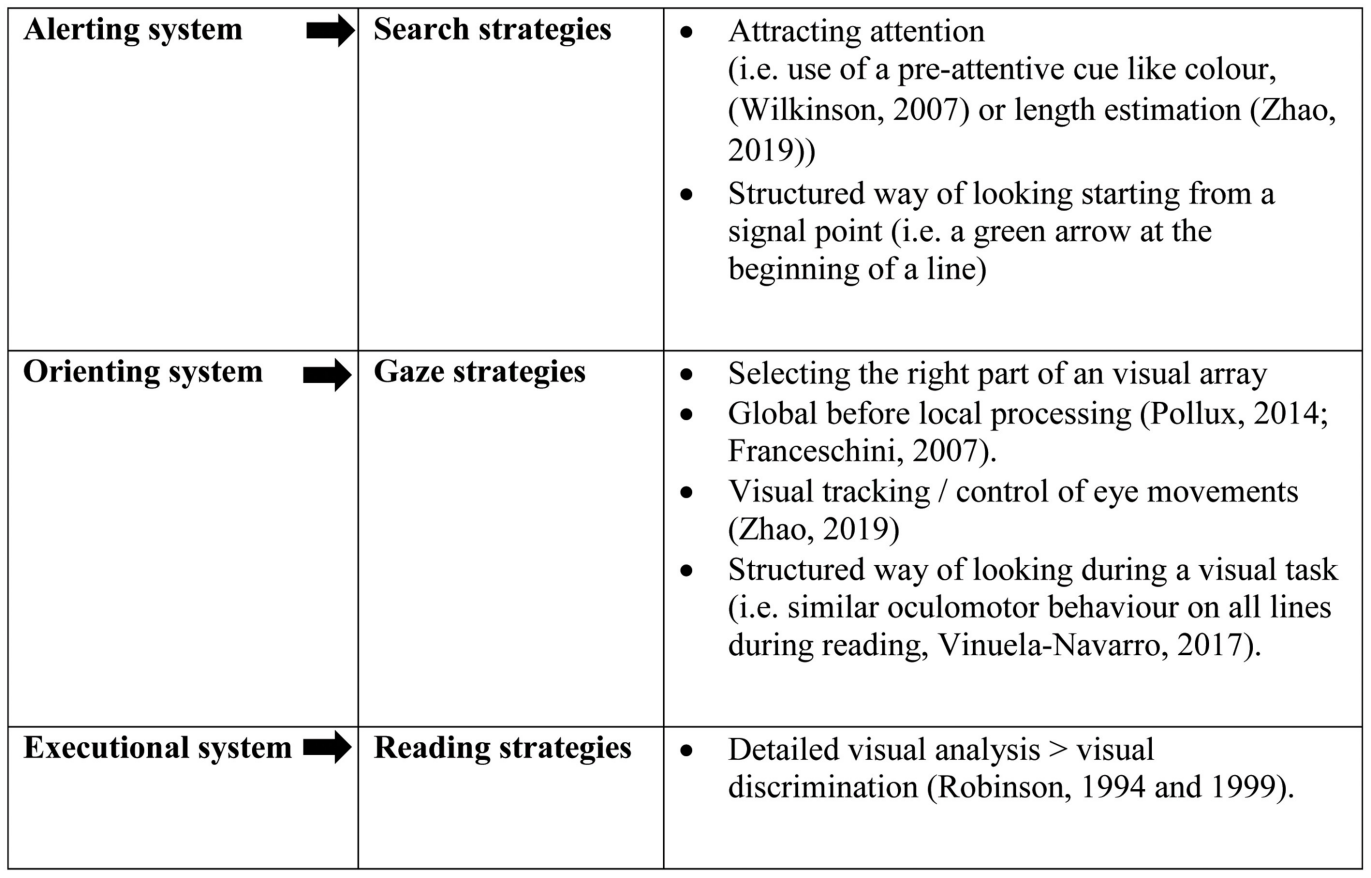
Viewing strategies in relation to Posner's attention networks.

A remarkable finding is that none of the five studies mentioning viewing strategies, address the role of attentional mechanisms. In the description of viewing strategies presented in Table 4, it is clear that the strategies are not restricted to one form of visual processing, but rather target the activation of attention systems. We have therefore connected the viewing strategies described in this review with the attention networks defined by Posner (see Figure 2, Petersen and Posner, 2012). For visual search, the strategy that was mentioned, was to use color cueing to facilitate visual search (Wilkinson et al., 2008). Color cueing might support the alerting network, because it can function as a pre-attentive signal to guide visual attention to a certain visual array (Wolfe and Utochkin, 2019). Using pop-out stimuli involves a sensory-driven or bottom-up mechanism directing perception toward a subset of stimuli, which might be useful strategy in children with attention deficits. For some children the use of colored overlays may help to arouse and/or sustain visual attention (Robinson and Conway, 1994). The opposite mechanism of bottom-up is top-down processing, which involves goal-directed mechanisms that are determined by the individual's goals (Donnelly et al., 2007). Top-down “guided” search strategies are more commonly used in conjunctive search tasks in which an individual has to look for a combination (i.e., consider color and shape). From an attention network point of view, top-down processing is especially dependent of executive network activity (Petersen and Posner, 2012). Executive network control improves during development and can be the aim of viewing strategy training. Gaze strategies refer to the part of the visual array which is processed, a strategy which can be linked to the orienting attention network. The orienting attention network enables prioritizing sensory input by selecting a modality or location, a process in which both frontal as well as parietal areas are implicated (Petersen and Posner, 2012). A holistic strategy appears to be helpful to extract all relevant visual cues from a visual stimulus, for example in recognizing facial expressions and reading (Pollux et al., 2014; Franceschini et al., 2017). Reading strategies can involve a structured way of moving the eyes through the lines of text (Vinuela-Navarro et al., 2017). Average readers turned out to show similar oculomotor behavior for each line of text and oculomotor functions seem to improve during primary school years (Medland et al., 2010). The analysis of oral reading errors by Robinson and Conway (1994) showed that a more detailed word analysis supported reading comprehension. Children with reading problems more often guessed words from single-letter cues, reread the lines and skipped words or lines. The executive network is involved in the visual discrimination of distinguishing visual features, i.e., letters during reading or search. We propose that viewing strategies which a child can use consciously and adjust to the task at hand, will support the executive network system.

The final goal of this scoping review was to identify possible interventions targeting viewing strategies that can improve visual processing. Due to the diversity in interventions and outcome measures, outcomes were presented in a narrative manner. We did not find any intervention studies targeting viewing strategies for children with (C)VI. Since improving viewing strategies is a common training goal in visual rehabilitation, more research concerning viewing strategies used by children with (C)VI is needed. Till date, it remains unknown whether there are differences in viewing strategies between children with normal vision and children with (C) VI. Also, even if school-aged children use the same kind of viewings strategies, it remains unclear if the quality of the viewing strategies differs between these groups, for example in speed of processing or accuracy of visual identification. Three studies showed lower visual processing speed in children with (C)VI (Kooiker et al., 2014, 2015; Barsingerhorn et al., 2018), although no relation with viewing strategies was investigated. In order to learn more about viewing strategies in typical and impaired childhood vision, future research should be directed at comparing spatial (gaze patterns) and temporal aspects of oculomotor behavior during task performance (e.g., reading and search).

Despite the lack of concrete measures in the field of viewing strategies, the included studies showed some starting points for the development of a training targeting viewing strategies in children with (C)VI. The influence of attentional processes on viewing behavior is shown in a variety of studies and stresses the importance of investigating the potential benefits of targeting task-relevant attention networks in visual training. VAS dysfunction, or more specific disorders in visual selective attention processes, can be one of the visual dysfunctions in children with CVI (Zuidhoek, 2020). VAS based training for children with dyslexia and VAS dysfunction showed improvements not only on visual attention span, but also on reading performance (Zhao et al., 2019). Including VAS based elements (e.g., length estimation task, digit canceling and visual tracking to train eye-movement control) in viewing strategy training could have an impact on attention network activity. Although eye movements appear to develop when children learn to read more fluently (Medland et al., 2010), training a structured way of moving the eyes over a line of text or images might improve reading speed as well. At the moment, it is unclear whether only practicing a viewing strategy like moving the eyes to specific spots on a line, is enough to contribute to improved attentional processes in children with (C)VI. For complex skills such as reading (especially in children with deficits in visual attentional processing), it is to be expected that enhancing “guided” top-down control, e.g. by consciously adopting a task-appropriate viewing strategy, could have a larger beneficial impact than oculomotor training.

Vision training targeting improvements in general visual processing, such as the “Frostig Visual Perception Training” or the “Learning to Look and Listen” program, showed ambiguous results (Bieger, 1974; Obrzut et al., 1982). Although children showed task-specific improvements after training, there was no learning transfer to educational measures. Perceptual learning, defined as a consistent change in the perception of a stimulus array following practice or experience with this array, resulted in improvements in a variety of visual functions, like near visual acuity, binocular vision and spatial aspects of reading in children with VI (Huurneman et al., 2013, 2016a,b,c, 2020; Yu et al., 2020). We did not find any intervention studies evaluating the effect of perceptual learning in children with CVI. Perceptual learning paradigms do not aim at the use of conscious viewing strategies, but lead to performance improvements on a perceptual task as a result of experience or repeated exposure to a task. Learning by perceptual experience differs from explicit top-down guided instruction to support visual processing. Traditional perceptual learning paradigms require a considerable amount of attention from a child. If there is a deficit in visual attention, the more logical first step in rehabilitation might be to learn the child to adopt viewing strategies that support visual attention.

In conclusion, this scoping review provides new leads toward the development of a viewing strategy training which can support visual attention processing. However, the specific relation between strategic viewing processes and academic performance remains unclear. A possible limitation of the current review is that the term ‘search strategies' was not included in the literature query. Including this term in the current review would widen the scope too much. Zhao et al. (2019) mentioned visual search as a top-down process when there was high target-distractor similarity. In visual search studies, both bottom-up as well as top-down processes are regularly described. For example, Donnelly et al. (2007) showed that targets with one unique feature (i.e., color or size) can be processed fast regardless of the amount of distractors (flat curve). If a target can only be distinguished based on a combination of features (i.e., conjunction search), then search time will increase as a function of distractor number. Interestingly, only conjunction search (and not feature search) efficiency seems to be affected by age: older children show shorter search times when the number of distractors increase (Donnelly et al., 2007). Future research is needed to examine the relation between the use and viewing strategies and visual performance and the role of development on the use of viewing strategies.

## Conclusions

This scoping review shows that, viewing strategies involved in daily school based tasks like reading, are a relatively new concept. We signaled a giant gap between current scientific literature and daily rehabilitation practice in which training programs aiming at viewing strategies are common. It remains unclear if and how viewing strategies of children with (cerebral) visual impairment differ from viewing strategies in children with normal vision. Clear definitions of viewing strategies were not found in the included studies. Gaze strategies, search strategies and reading strategies were described as elements contributing to visual processing. The use of viewing strategies might influence different visual attention networks (i.e., the alerting, orienting and executive network). We propose that for the development of an effective viewing strategy, the role of these three networks should be considered. Although the scoping review revealed attentional processes that are involved in viewing behavior, we did not find any interventions that targeted visual attentional processing in children with (cerebral) visual impairment. The relation between strategic viewing processes and academic performance therefore remains unclear.

## Data Availability Statement

The original contributions presented in the study are included in the article/Supplementary Material, further inquiries can be directed to the corresponding author.

## Author Contributions

Literature screening and selection, data extraction, and synthesis was performed by AF-V and BH. Preparation of the first draft of the manuscript was done by AF-V. Review and approval of the manuscript was performed by BH and FB. All authors read and approved the final manuscript.

## Funding

This research was supported by Novum. The funder was not involved in the study design, collection, analysis, interpretation of data, the writing of this article or the decision to submit it for publication.

## Conflict of Interest

The authors declare that the research was conducted in the absence of any commercial or financial relationships that could be construed as a potential conflict of interest.

## Publisher's Note

All claims expressed in this article are solely those of the authors and do not necessarily represent those of their affiliated organizations, or those of the publisher, the editors and the reviewers. Any product that may be evaluated in this article, or claim that may be made by its manufacturer, is not guaranteed or endorsed by the publisher.
