# Supplementary material for: Viewing Strategies in Children With Visual Impairment and Children With Normal Vision: A Systematic Scoping Review
Source: Front Psychol. 2022 Jun 17;13:898719. doi: 10.3389/fpsyg.2022.898719 (PMC9248372; doi:10.3389/fpsyg.2022.898719)
Supplement: Supplementary file 1 [file Data_Sheet_1.pdf]

**Supplemental Table 1.** Study characteristics for included studies on viewing behaviour in children with normal vision (n =14).

| Reference | Type of study | Number of participants, group | Age | Method | Definition viewing strategy |
|---|---|---|---|---|---|
| Benfatto 2016 | Longitudinal cohort | N = 97 high risk children for dyslexia<br>N = 88 low risk subjects (LR) | 9-10 years | Evaluation of oculomotor measures collected during reading. | No |
| Eden 1995 | Case control | N= 39 normal controls<br>N= 26 reading disabled children (RD)<br>N=12 backward reading children (BR) | 10-11 years | Performance on temporal and spatial dot counting task. | No |
| Fisher 1985 | Cross sectional | N = 50, kindergarten<br>N = 50, 1st grade | 4-7 years | Left-right coding, letter production and (beginning reading skills). | No |
| Franceschini 2017 | Case control | Experiment 1<br>N = 180 (dyslectic N = 17, mean age 9.17; Normal readers N = 162)<br>Experiment 2<br>N=13 dyslectic<br>Experiment 3<br>N = 32 dyslectic; N = 15 normal readers)<br>Experiment 4<br>N = 14 dyslectic, mean age 10.41)<br>Experiment 5<br>N = 96 five year old children / T2 N = 82) | 5-11 Years | NAVON task and reading tasks | No |
| Garcia 2019 | Case control | Study 1<br>N = 28 severe reading difficulties (RD),<br>N = 28 controls<br><br>Study 2 | 9 – 10 years | Nonsense shapes and non-words (4 phonemes) in fixed and variable bindings during a working memory test. | No |

| | | | | | |
|---|---|---|---|---|---|
| | | N = 28 RD (new sample)<br>N = 28 controls | | | |
| Lutzer<br>1986 | Cross sectional | Four groups of children:<br>- Children with mental impairment (8 year olds), N = 15<br>- Average 8-year-old group, N = 24<br>- Gifted 6-year-old group, N = 24<br>- Average 6-year-old group, N = 24 | 6 – 8 years | A colour discrimination task was used with match-to-sample or preference-ranking training procedures.<br><br>Outcome measures:<br>• Sum of correct responses within each six-trial training or test in which a match-to-sample task was used. | No |
| Medland<br>2010 | Cross sectional | <u>English readers</u><br>Children, N = 43<br>Adults, N = 20<br><br><u>Arabic readers</u><br>Children, N = 6<br>Adults, N = 5 | 5 – 11 years | Developmental Eye Movements (DEM) test. | No |
| Perea<br>2015 | Cross sectional | N = 20 children | 4 years | Same-different matching experiment in preliterate 4-year-old children using same versus different trial (created by letter transposition (TZ-ZT) or replacement (GC-GX)). | No |

| | | | | | |
|---|---|---|---|---|---|
| Riddell 1990 | Cross sectional | Group 1<br>• Children referred for reading difficulties, N = 50 (group 1)<br><br>Group 2<br>• Children tested at Ascot Heath Infant School, N = 81 (group 2)<br><br>Children with visual or overt oculomotor impairments were excluded. | Group 1: 6 – 9 years<br><br>Group 2: 4 – 6 years | Comparison of the accuracy of spatial localisation on a non-linguistic computer game by children having good and poor vergence control on Dunlop test.<br><br>Outcome measures: Dot-localisation task: percentage of errors. | No |
| Solan 2007 | Case control | N = 19 good readers<br><br>N = 23 poor readers | Grade 7 students (mean age 11 years) | - visual attention skills (Cognitive Assessment System (CAS))<br>- magnocellular integrity (Coherent Motion Threshold (CM))<br>- reading skills (Gates-MacGinitie Reading Tests, reading comprehension subtest) | No |
| Tong 2019 | Cross sectional | N = 35 children with developmental dyslexia<br>N = 37 chronologically age-matched controls | 7-8 years | - Visual statistical learning (triplet learning paradigm based on the study by Arciuli and Simpson, 2011)<br>- Orthographic awareness<br>- Chinese word reading (150 two-character word list) | No |

| | | | | | |
|---|---|---|---|---|---|
| | | | | - Word dictation tasks<br>- Nonverbal cognitive ability (Raven's Progressive Matrices, set A and B) was used as a covariate for the two groups | |
| Vagge 2015 | Case control | N = 11 children with dyslexia<br>N = 11 controls | 8 – 13 years | Eye movement analysis during reading a text | No |
| Vinuela-Navarro 2017 | Case control | N = 120 children without delayed reading skills (4-11y)<br>N = 43 children with delayed reading skills (4-11y) | 4 – 11 years | Saccade measures, main sequence (collected with the Tobii TX300 eye tracker by showing children cartoon characters horizontally from -20° to +20° in steps of 5°). Fixation stability (by showing children an animated stimulus in the centre of the screen for 8sec). Saccade number and amplitude during fixation | Yes, reference to Lefton (2014): good readers showed a similar eye movement strategy for each line of text during reading (number of saccades, fixations and duration of fixations were comparable), whereas poor readers performed very differently in each line (unstructured and disorganised eye movement strategy) |

| | | | | | |
|---|---|---|---|---|---|
| Wilkinson 2008 | Cross sectional | N = 10 children with Down Syndrome (DS)<br><br>N = 8 typically developing children > 4 years (TDO)<br><br>N = 8 typically developing children < 4 years (TDY) | DS: 106 – 201 months;<br><br>TDO: 48 – 57 months;<br><br>TDY: 40 – 46 months; | Line drawings of PCS symbols in two colour conditions (clustered and distributed arrays). Three tasks: auditory – visual matching of food stimuli, visual - visual matching of clothing stimuli, visual – visual matching of activity stimuli.<br>Accuracy (percentage correct) and speed (RT). | Yes, search strategy: colour cueing facilitates visual search for symbols |

**Supplemental Table 2.** Study characteristics for included studies on viewing behaviour in children with (cerebral) visual impairment (n = 3).

| Reference | Type of study | Number of participants, group | Age | Method | Definition viewing strategy |
|---|---|---|---|---|---|
| Barsingerhorn 2018 | Cross sectional | N = 30 children with visual impairments due to ocular dysfunction<br><br>N = 17 children with CVI | 5 – 12 years | Speed acuity test regarding orientation of Landolt C symbols: RT for symbols ranging between -0.3 and 1.2 LogMAR relative to acuity threshold compared to normative data.<br>RT to Visual Detection Task (VDT) compared to normative data.<br>RT to Auditory Detection Task (ADT) compared to normative data. | No |
| Kooiker 2014 | Cross sectional | Children with (suspected) visual information processing impairments ('at risk group', N = 149)<br><br>Children with typical visual development (N = 127) | 1 – 13 years | Measurement of visual orienting responses collected during presentation of cartoon stimuli (cartoons were shown 32 times, whereas other stimuli were shown 8 times, presentation time 4sec). Stimuli were presented in specific areas in one of the quadrants of the monitor (target area).<br>Outcome measure: Reaction time to fixation on a stimulus (RTF). | No |
| Kooiker 2015 | Cross sectional | Children attending special education for the visually impaired (N = 104) | 1 – 12 years | Examination of the relation between orienting responses and factors associated with visual processing impairments in clinical practice (i.e. gender, prematurity, clinical diagnosis CVI, epilepsy, nystagmus, strabismus, ocular motor abnormalities, visual field defect, behavioural disorder, perceptual dysfunction, age, visual acuity, level of intelligence).<br><br>Outcome measures: RTF and fixation quality | No |

**Supplemental Table 3.** Study characteristics for included studies on interventions studies targeting viewing behaviour (n = 13).

| Reference | Type of study | Number of participants, group | Age | Method | Definition viewing strategy |
|---|---|---|---|---|---|
| Bieger 1974 | Non-randomized controlled trial (non – RCT) | Children with (assumed) normal intelligence with specific reading disabilities, divided into two groups:<br><br>• N = 25 experimental group (remedial teaching (RT) + Frostig Visual Perception Training program, 8 months 2/week)<br>• N = 23 Control group (RT) | 6;10 – 8;9 years | Pre- and post-measures:<br>• Frostig Perceptual Quotients<br>• Durrell Test of Visual Discrimination of Words<br>• Durrell Identifying Lower Case Letters<br>• Spache Diagnostic Reading Scales | No |
| Hall 2013 | Non-RCT | Children with delayed reading skills, divided into two groups:<br><br>• N = 37 Dyslexia Reading Trust (DRT): yellow/blue filter<br>• N = 36, Harris group: optimal colour filter for each eye | DRT group 9;8y±(SD)9.5 months<br><br>Harris group 9;1y±12.6 months | Pre- and post-tests (without filters):<br>• British Ability Scales (BAS) II reading and spelling<br>• Time per correct word on the Castles and Coltheart's lists. | No |
| Huurneman 2013 | Non-RCT | Children with visual impairment (N = 45), divided into three groups:<br><br>• Magnifier group, N = 12 | 4-9 years | Children with VI trained 2× per week for a period of six weeks (12 training sessions, 30 min. each). Children trained under supervision at school. | No |

| | | | | | |
|---|---|---|---|---|---|
| | | • Pen-and-paper crowded perceptual learning (PL) group, N = 18<br>• Pen-and-paper uncrowded PL group, N = 15<br><br>Children with normal vision (N = 29, no training) | | Outcome measures:<br>• Single and crowded near visual acuity<br>• Crowding ratio<br>• Number of trials<br>• Accuracy<br>• Performance time<br>• Small errors (incorrect drawing one noninversed E)<br>• Large errors (incorrect drawing > 1 noninversed E) | |
| Huurneman 2016 | Non-RCT | Children with infantile nystagmus (IN, n = 36), divided into two groups:<br><br>• Computerized uncrowded letter training (N = 18)<br>• Computerized crowded letter training (N = 18)<br><br>Training occurred 2× per week during 5 weeks under supervision at school.<br><br>Children with normal vision (N = 11, no training) | 6-11 years | Outcome measures:<br>• Nystagmus characteristics (amplitude, frequency, intensity, expanded nystagmus acuity function (NAFX))<br>• Fixation stability<br>• Saccadic eye movements | No |
| Huurneman 2016_2 | Non-RCT | See above (Huurneman 2016) | 6-11 years | Outcome measures:<br>• Reading acuity<br>• Maximum reading speed (wpm) | No |

| | | | | | |
|---|---|---|---|---|---|
| | | | | • Critical print size<br>• Acuity reserve | |
| Huurneman 2016_3 | Non-RCT | See above (Huurneman 2016) | 6-11 years | Outcome measures:<br>• Task-specific performance<br>• Distance and near visual acuity<br>• Intensity and extent of crowding<br>• Stereopsis | No |
| Huurneman 2020 | Non-RCT | Children with VI (N = 16), divided into two groups:<br><br>• Early treatment group (N = 9)<br>• Late treatment group (N = 7) | 4-8 years | Children trained 2× per week for a period of 6 weeks (12 sessions) with a pen-and-paper drawing training based on perceptual learning principles. Children in the early treatment group were measured 4 times since inclusion and children in the late treatment group were measured 5 times since inclusion.<br><br>Outcome measures:<br>• Uncrowded and crowded NVA<br>• Uncrowded and crowded DVA<br>• Beery VMI, subtest Motor Control | No |
| Orbutz 1982 | Non-RCT | Children with poor visual processing skills (n = 153), divided into three groups:<br><br>• Training group (N = 51)<br>• Contrast group (N = 51)<br>• Control group (N = 51) | Kindergarten first and second graders (5-8 years) | Training group = a standardized visual information processing training program, Learning to Look and Listen, was used. Training took place daily (25 minutes each day) during 6-7 weeks. Supervision was given to groups of 4-6 children. | No |

| | | | | | |
|---|---|---|---|---|---|
| | | | | Contrast group = taught in groups of 4-6 children who were given instruction, using regular curriculum material in a variety of tasks such as colouring, cutting, storytelling, but was not given specific visual training.<br><br>Control group = remained in regular classroom and school program.<br><br>Outcome measures (pre, post, and 6w follow up):<br>• MFFT<br>• Bender Gestalt Test (BGT)<br>• Boehm Test of Basic Concepts (BTBC)<br>• Metropolitan Achievement Tests (MAT) | |
| Pollux 2014 | Cohort study | Children with normal vision (N = 16)<br><br>Adults with normal vision (N = 16, not further regarded) | 8;2-9;3 years | Four training sessions on four consecutive days with a self-paced, free-viewing facial expression categorization task using emotional faces with varying intensity levels.<br><br>Outcome measures:<br>• Behavioural measures (accuracy, response times, incorrect response)<br>• Eye movement measures (number of fixations, proportion of fixations and viewing times on different facial | Yes, gaze strategy: a holistic viewing strategy is used to extract relevant facial cues from all internal features when categorizing subtle expressions. |

| | | | | | |
|---|---|---|---|---|---|
| | | | | features, proportion of fixations and viewing times of different facial features during second fixation) | |
| Robinson 1994 | Non-RCT | Experimental group<br>N = 29 children with reading or study problems<br><br>Control group<br>N = 31 children with similar reading and learning problems as the experimental group (age matched) | 9-14 years | Questionnaire relating to reading and writing performance, series of visual tasks and assessment whether performance is influenced by color overlays. | Yes, reading strategies mentioned:<br>-guessing words from single-letter cues<br>-rereading of lines<br>-skipping words or lines |
| Robinson 1999 | Non-RCT | Experimental group<br>N = 113 children with reading<br><br>Control group<br>N = 35 children with children with reading difficulties (age matched) | 9-13 years | Questionnaire relating to reading and writing performance, series of visual tasks and assessment whether performance is influenced by color overlays after 20-month use. | Yes, see above |
| Yu 2020 | Non-RCT | Children with VI (N = 28), divided into two groups:<br><br>• PL under assistance of electronic visual aid (EVA) (N = 14)<br>• Simple PL without EVA (N = 14) | 6-14 years | Training was given 30 minutes per day for 6 months. The EVA could provide 5-10x magnification on the 4.3-inch screen.<br><br>Outcome measures:<br>• Uncrowded distance VA<br>• Best corrected VA<br>• Near visual acuity | No |

| | | | | | |
|---|---|---|---|---|---|
| | | | | • Refractive error | |
| Zhao 2019 | Non-RCT | N = 10 trained dyslexic children with visual attention span (VAS) deficits<br><br>N = 10 untrained dyslexic children with VAS dysfunction<br><br>N = 10 trained dyslexic individuals with intact VAS<br><br>N = 10 untrained individuals with intact VAS<br><br>N = 14 age-matched normal readers | 10 years | VAS based training tasks (10 training sessions, given over a period of four weeks) including:<br><br>- length estimation task regarding bottom-up attention<br>- visual search and digit cancelling tasks targeting top-down attentional modulation<br>- visual tracking tasks to train eye-movement control<br><br>Outcome measures (pre- and post VAS-based training):<br>- Reading measures<br>-Visual 1-back test | No |

**Supplemental Table 4a.** Risk of bias assessment: QUADAS-2 Assessment for non-intervention studies. Criteria were scored as yes (+), no (-) or unknown (?).

| | Were selection criteria clearly described? | Was the execution of the test procedure described in sufficient detail to permit replication of the test? | Were uninterpretable/ intermediate test results reported? | Were withdrawals from the study explained? |
|---|---|---|---|---|
| Barsingerhorn 2018 | + | + | - | n.a. |
| Benfatto 2016 | + | + | - | n.a. |
| Eden 1995 | + | + | - | n.a. |
| Fisher 1985 | - | + | - | - |
| Franceschini 2017 | + | - | - | n.a. |
| Garcia 2019 | + | + | - | n.a. |
| Kooiker 2014 | + | + | - | + |
| Kooiker 2015 | + | + | - | + |
| Lutzer 1986 | - | + | - | n.a. |
| Medland 2010 | - | + | - | + |
| Perea 2015 | + | + | - | n.a. |
| Riddell 1990 | + | + | - | n.a. |
| Solan 2007 | + | + | - | n.a. |
| Tong 2019 | + | + | - | n.a. |
| Vagge 2015 | + | ? | - | n.a. |
| Vinuela-Navarro 2017 | + | + | - | + |
| Wilkinson 2008 | + | + | - | + |

**Supplemental Table 4b.** Risk of bias assessment: Cochrane Assessment for intervention studies**.** Criteria were scored as high risk (+), low risk (-) or unknown (?).

| | Selection bias | Performance bias | Detection bias | Attribution bias | Reporting bias |
|---|---|---|---|---|---|
| Bieger 1974 | + | - | - | ? | - |
| Hall 2013 | - | - | - | - | - |
| Huurneman 2013 | + | ? | + | - | - |
| Huurneman 2016 | - | ? | + | - | - |
| Huurneman 2016_2 | - | ? | + | - | - |
| Huurneman 2016_3 | - | ? | + | - | - |
| Huurneman 2020 | - | ? | + | - | - |
| Orbutz 1982 | - | + | ? | - | - |
| Pollux 2014 | + | + | + | - | - |
| Robinson 1994 | + | - | - | - | - |
| Robinson 1999 | - | ? | - | - | - |
| Yu 2020 | + | ? | ? | - | - |
| Zhao 2019 | - | ? | ? | - | - |
